# Optimal linguistic expression in negotiations depends on visual appearance

**DOI:** 10.1371/journal.pone.0195496

**Published:** 2018-04-05

**Authors:** Maki Sakamoto, Jinhwan Kwon, Hikaru Tamada, Yumi Hirahara

**Affiliations:** 1 Department of Informatics, The University of Electro-Communications, Tokyo, Japan; 2 Pallas Global Enterprise, Tokyo, Japan; Kyoto University, JAPAN

## Abstract

We investigate the influence of the visual appearance of a negotiator on persuasiveness within the context of negotiations. Psychological experiments were conducted to quantitatively analyze the relationship between visual appearance and the use of language. Male and female participants were shown three female and male photographs, respectively. They were asked to report how they felt about each photograph using a seven-point semantic differential (SD) scale for six affective factors (positive impression, extraversion, intelligence, conscientiousness, emotional stability, and agreeableness). Participants then answered how they felt about each negotiation scenario (they were presented with pictures and a situation combined with negotiation sentences) using a seven-point SD scale for seven affective factors (positive impression, extraversion, intelligence, conscientiousness, emotional stability, agreeableness, and degree of persuasion). Two experiments were conducted using different participant groups depending on the negotiation situations. Photographs with good or bad appearances were found to show high or low degrees of persuasion, respectively. A multiple regression equation was obtained, indicating the importance of the three language factors (euphemistic, honorific, and sympathy expressions) to impressions made during negotiation. The result shows that there are optimal negotiation sentences based on various negotiation factors, such as visual appearance and use of language. For example, persons with good appearance might worsen their impression during negotiations by using certain language, although their initial impression was positive, and persons with bad appearance could effectively improve their impressions in negotiations through their use of language, although the final impressions of their negotiation counterpart might still be more negative than those for persons with good appearance. In contrast, the impressions made by persons of normal appearance were not easily affected by their use of language. The results of the present study have significant implications for future studies of effective negotiation strategies considering visual appearance as well as gender.

## Introduction

Our first impressions of others are often based on the visual appearance of their faces [1]. We tend to evaluate others based on their appearance and then move on to interact with them based on these first impressions [2]. For example, humans are said to be excellent in judging personalities and complex social characteristics based on appearance, such as dominance, hierarchy, warmth, and threat [3][4][5][6][7][8]. People often rely on their emotions or subjective impressions, either intentionally or unintentionally, to shape a wide variety of judgements including social, political, and personal decisions [9]. Emotions have been studied extensively in the domain of persuasion [10]. According to previous studies, a person’s emotions, whether stemming from a persuasive message or contextual factors, can influence evaluative judgements through multiple cognitive processes (for a review, see [11]. In the present study, we address how the relationship between persuasiveness and visual appearance, as well as the use of language, affects emotions.

Previous studies of visual appearance have suggested that there exists a stereotype associated with physical attractiveness referred to as “what is beautiful is good” (for more reviews, see [12][13] [14][15]). For example, [12] found that strangers rated attractive people as possessing socially desirable traits to a greater extent than unattractive people. Several studies examining this attractiveness stereotype have demonstrated that attractive people are seen in a positive light for a wide range of attributes compared with unattractive people (although some negative attributes, such as vanity, are attributed to attractive individuals ([16][17]; for a review [18]) In our study, we reconsider this popular conception that “what is beautiful is good.” In doing so, we created attractive and unattractive male and female faces for evaluation by different genders because first impression and perceptual attributions to facial photographs are reported to be important in partner choice by both genders [19][20][21]. Our experiments focus on the consistency of the first impression made by facial attractiveness in the negotiation process. [22] presents a general framework of the role of emotion in the negotiation process. In the prenegotiation step, interpersonal attractiveness may contribute to the formation of positive prenegotiation affect, but initial expectations will be amended to the extent that they are disconfirmed. That is, negative violations of expectancies will lower such expectations as the negotiation proceeds, while positive violations will raise expectations. In our study, we analyze how the first impressions made from facial attractiveness will change during the negotiation process.

We focus on the use of language as a potential factor influencing the negotiator’s impressions. Language is highly important during negotiation. Many studies in sociolinguistics have investigated the relation between linguistic styles and impression formation. [23] argued the relationship between speech style and person perception and persuasion processes based on experimental results, which showed that use of a powerful linguistic style marked by less frequent use of intensifiers, hedges, hesitation forms, and questioning intonations resulted in greater perceived credibility of the witness than did the powerless linguistic style. Previous social psychological research on the effects of speech style has generally involved the manipulation of linguistic variables [24][25]. Therefore, we, compare the effects of different linguistic styles on evaluation of the speaker. The use of particular speech styles depends on the specific situation within the speech occurs [23]. Thus, we also compare the effects of different linguistic styles on evaluation of the speaker across different situations.

According to previous studies, speech style is related to variables such as the speaker’s gender [26][27], social class, and ethnic group [28]. Many of the linguistic features that distinguish powerless from powerful speech have been hypothesized to show gender differences in language use [29][30][31]. In our study, we pay attention to the gender difference of the effects of linguistic styles on evaluation of the speaker because the Japanese language tends to convey masculinity or femininity through linguistic expressions such as sentence endings [32] [33]. Gender stereotypes are a cross-cultural matter. [34][35] conducted a large-scale, cross-cultural study of gender stereotypes in 25 countries from Europe, Asia, Africa, Oceania, and the Americas. Their results showed that in all countries, adjectives associated with men were stronger and more active than the adjectives associated with women. Such gender stereotypes are closely linked to traditional social roles and power inequalities between women and men [36]. Early studies of gender-role identity revealed these stereotypes [37]. [37] asked male and female participants to rate each of a large pool of traits in terms of desirability for a woman or man. The criteria yielded the 20 feminine and 20 masculine characteristics that appear on the Bem Sex-Role Inventory (for a review, see [38]). Feminine characteristics are: affectionate, cheerful, childlike, compassionate, does not use harsh language, feminine, gentle, loyal, sensitive to the needs of others, shy, soft-spoken, sympathetic, tender, understanding, and warm. These characteristics are not desired for men (for more details of gender stereotypes [39]). Gender stereotypes may be related to the effects of different linguistic styles on evaluation of female and male speakers. We analyze whether desirable speech style differs between female and male speakers.

The main objective of this study was to investigate how visual appearance affects the persuasiveness of the negotiation process. Previous studies have argued that people tend to evaluate others based on their appearance and interact with them based on their first impressions. Here, we want to reconsider the stereotype, “what is beautiful is good.” Furthermore, the secondary question is to examine how the use of language according to visual appearance affects affective factors during the negotiation process and the influence of gender. Previous sociolinguistics studies have investigated the relation between linguistic styles and impression formation but have not focused on the influence of visual appearance. We created three types of female and male photographs and asked participants to report how they felt about each photograph using a seven-point semantic differential (SD) scale for six affective factors (positive impression, extraversion, intelligence, conscientiousness, emotional stability, and agreeableness). In addition, we created negotiation scenarios to examine how participants felt about each negotiation scenario (they were presented with pictures and a situation combined with negotiation sentences) using a seven-point SD scale for seven affective factors (positive impression, extraversion, intelligence, conscientiousness, emotional stability, agreeableness, and degree of persuasion).

## Materials and methods

### Ethics statement

This study was approved by the Research Ethics Committee of the University of Electro-Communications, Tokyo, Japan. The study adhered to the tenets of the Declaration of Helsinki (2013). All participants provided written informed consent prior to the experiment. Documents explaining the experimental procedures and the written informed consent forms were presented to the ethics committee.

### Participants

The participants in Experiment 1 were 66 university students (33 women, 33 men) aged between 20 and 25 years (mean = 21.74, standard deviation [S.D.] = 1.15). The participants in Experiment 2 were 60 married, working participants (30 women, 30 men) aged between 20 and 64 years (mean = 46.97, S.D. = 9.81). All participants had normal hearing and normal or corrected-to-normal visual acuity, and were naïve as to the purpose of the experiment. Participants were paid to take part in the experiments.

### Photographs

We used three female and three male photographs, which were edited into three groups of photographs to represent three levels of physical attractiveness, respectively. Public domain photographs of one woman and one man were taken from the Internet. Using the online photo editor Pixlr (https://pixlr.com/editor/), we transformed these photographs into images that were more and less physically attractive (i.e., more and less cute). We made seven morphed photographs by transforming the hairstyle, eyes, nose, mouth, and facial form from the original male/female photographs. The original for each photograph was not included in the seven photographs. The authors and sociology experts selected three photographs by self-examination; we then asked 20 university students (10 women, 10 men) to categorize the six photographs into cute, normal, and not-cute. We confirmed that there was a significant difference in the attractiveness of the first impression of the three photographs from the evaluation experiment (F(2, 5) = 73.851, *p* < .001).

### Negotiation scenarios

Because the effects of linguistic styles depend on the specific situation within which the speech occurs [23], we created various situations in which the persons in the photographs negotiated with the participants, who acted as their counterparts. The four negotiation situations for female participants cast the negotiators in the photographs as their boyfriend, husband, male colleague, or male superior, while the four negotiation situations for male participants cast the female negotiators in the photographs as their girlfriend, wife, female colleague, or female superior. We created three patterns of negotiation sentences for each of the four negotiation situations (see Table 1). These 12 negotiation sentences were constructed to include euphemistic, honorific, and sympathy expressions. These expressions are believed to be important for negotiations in the Japanese language, and are especially important for female negotiators, who are expected to fit the previously listed female stereotypes. We then created variations of these basic 12 sentences, including those with only euphemistic expressions, those including only honorific expressions, those including only sympathy expressions, those including euphemistic expressions and honorific expressions, those including euphemistic expressions and sympathy expressions, and those including honorific expressions and sympathy expressions. As a result, we had 84 negotiation sentences (12 basic sentences and 72 variations).

**Table 1:** Basic negotiation sentences.

Negotiator	Negotiation sentences
Subordinate	The project team which you lead has been asked to give a last-minute presentation at the next planning meeting to take place in 2 days.
	I understand you want to give the presentation at this week’s meeting. (sympathy) However that would require overloading everyone with more work than they can handle. Could we also consider passing up this week's meeting and preparing instead for next week's meeting? (euphemistic) Perhaps our proposal will be better received if we prepared for it more thoroughly, what do you think? (euphemistic)
Subordinate	Between projects A and B, you have selected project A as most appropriate for this subordinate.
	Thank you for selecting me for project A. I intend to fully live up to your expectations. (sympathy) But considering the work descriptions and my own strengths, I feel that perhaps I may be able to contribute more to project B. (euphemistic) Would it be possible to ask you to reconsider my assignment? (euphemistic)
Subordinate	You have just asked your subordinate to prepare some documents.
	About the documents you requested, unless you need them immediately (sympathy), do you think it would be permissible for me to take my lunch first? (euphemistic) I will prepare them when I return and I can get them to you by 2 pm.
Colleague	I realize that you are very busy (sympathy), but could I possibly ask you to mail these documents to __Corp.? (euphemistic) They need to be sent right away, to arrive tomorrow, but I’m just heading out the door. I’d be so happy if you could help me out! (sympathy)
Colleague	I’m sorry to bother you (sympathy), but could I trouble you to give me some advice on this report? (euphemistic) It’s due next week, and I’m having a hard time putting it together.
Colleague	You haven’t finished preparing the documents that your colleague asked for earlier.
	I realize that you’re busy (sympathy), and I don't mean to pressure you (sympathy), but would it be possible to have those documents done soon? (euphemistic)
Boyfriend/girlfriend	You are attending a party.
	I’m glad I got to meet you! (sympathy) We have a lot in common and the time flew by. If you wouldn’t mind, and if you have time (sympathy), would you like to have dinner with me? (euphemistic) I’d love to hear more of your stories!
Boyfriend/girlfriend	You are having a conversation about a newly built theme park.
	About that theme park, if we could go together, that would be so much fun! (sympathy) Perhaps you could invite me along next time you go? (euphemistic)
Boyfriend/girlfriend	You’re looking forward to a completely free weekend, as it’s been a while since you had one.
	I’m going to a friend’s wedding this weekend, and they want everyone to bring a date for the reception party. I’d be so thrilled if you could go with me! (sympathy) Would you please think about it? (euphemistic)
Husband/wife	You are not particularly interested in the opera.
	My colleague at work gave me two tickets to the opera! I know you’re not into the opera, but I can’t think of anyone else I’d prefer to go with. (sympathy) Would it be asking too much to ask you to go with me? (euphemistic)
Husband/wife	A request from your stay-at-home wife/husband.
	Remember I mentioned that my friend ____ is having a party next month? If you wouldn’t mind terribly (sympathy), would it be alright if I spent about 50,000 yen on a nice suit for the party? (euphemistic)
Husband/ wife	You are planning to go out for drinks with your colleagues after work.
	If perhaps you don’t have too much luggage this evening (sympathy), would it be alright to ask you to stop at the supermarket on your way home? (euphemistic) I’d be really happy if you could! (sympathy) I’ve put it all on this list. Please?

Default expressions are honorific. Euphemistic expressions are underlined. Sympathy expressions are double underlined.

### Task and procedure

[40] described the experiment using female photographs in detail. This paper compares the experimental results using male photographs with the previous results [40] results; therefore, we describe our procedure with reference to the previous study. We asked the participants to report how they felt about each photograph using a seven-point SD scale for six affective factors (positive impression, extraversion, intelligence, conscientiousness, emotional stability, and agreeableness). They then answered how they felt about each negotiation scenario (they were presented with pictures and a situation combined with negotiation sentences) using a seven-point SD scale for seven affective factors (positive impression, extraversion, intelligence, conscientiousness, emotional stability, agreeableness, and degree of persuasion). This procedure was conducted in two experiments using different participant groups depending on the negotiation situations.

The participants in Experiment A were 66 paid university students (33 women, 33 men) aged between 20 and 25 years (mean = 21.74, S.D. = 1.15). First, female group A (16 female participants) evaluated their impressions of the three male photographs and female group B (17 female participants) evaluated their impressions of the three female photographs. In addition, male group A (17 male participants) evaluated their impressions of the three female photographs and male group B (16 male participants) evaluated their impressions of the three male photographs. We then asked the participants in Experiment A to evaluate their impressions of negotiation sentences delivered by a boyfriend or girlfriend and by a colleague. For the negotiation sentence evaluation task, female and male participants were separated into two groups by gender to reduce their load. Each group was assigned half of the negotiation sentences. Half of female group A was assigned to 36 boyfriend negotiation sentences and 27 male colleague negotiation sentences. Half of female group A was assigned to 27 boyfriend negotiation sentences and 36 male colleague negotiation sentences. Half of female group B was assigned to 36 female colleague negotiation sentences. Half of female group B was assigned to 27 female colleague negotiation sentences. Half of male group A was assigned to 36 girlfriend negotiation sentences and 27 female colleague negotiation sentences. Half of male group A was assigned to 27 girlfriend negotiation sentences and 36 female colleague negotiation sentences. Half of male group B was assigned to 36 male colleague negotiation sentences and half of male group B was assigned to 27 male colleague negotiation sentences.

The participants in Experiment B were 60 paid, married, and working participants (30 women, 30 men) aged between 20 and 60 years (mean = 446.97, S.D. = 9.81). First, female group A (15 female participants) evaluated their impressions of the three male photographs and female group B (15 female participants) evaluated their impressions of the three female photographs. In addition, male group A (15 male participants) evaluated their impressions of the three female photographs and male group B (15 male participants) evaluated their impressions of the three male photographs. We asked the participants in Experiment B to evaluate their impressions of negotiation sentences delivered by a husband or wife and by a superior. For the negotiation sentences evaluation task, female and male participants were separated into two groups by gender to reduce their load and each group was assigned to half of the negotiation sentences. Half of female group A was assigned to 36 husband negotiation sentences and 27 male superior negotiation sentences. Half of female group A was assigned to 27 husband negotiation sentences and 36 male superior negotiation sentences. Half of female group B was assigned to 27 female superior negotiation sentences. Half of female group B was assigned to 36 female superior negotiation sentences. Half of male group A was assigned to 36 wife negotiation sentences and 27 female superior negotiation sentences. Half of male group A was assigned to 27 wife negotiation sentences by wife and 36 female superior negotiation sentences. Half of male group B was assigned to 36 male superior negotiation sentences and half of male group A was assigned to 27 male superior negotiation sentences.

## Results

As a result of experiments A and B, we obtained 29,106 items of data (66 patterns of negotiation stimuli ×7 SD scales ×63 participants). First, we examined the degrees of persuasion using three photographs. One-way factorial analysis of variance (ANOVA) of the degrees of persuasion showed a significant main effect for visual appearance (F(2, 5949) = 455.35, *p* < .001). Multiple comparisons with Bonferroni correction showed significant differences between appearances A and B (*p* < .001), between appearances A and C (*p* < .001), and between appearances B and C (*p* < .001). In particular, photographs with good appearance resulted in a high degree of persuasion and photographs with bad appearance resulted in a low degree of persuasion.

Furthermore, we used multiple regression to analyze how the use of language according to visual appearance affects affective factors. In particular, we focused on the standardized partial regression coefficient to confirm whether optimal linguistic expressions might be different depending on the negotiator’s appearance.

Regression analysis was conducted for all data to determine a general tendency for how euphemistic, honorific, and sympathy expressions affect seven impression scales (positive impression, extraversion, intelligence, conscientiousness, emotional stability, agreeableness, and degree of persuasion). Using the average of the rating values of the seven impression scales as objective variables and the variation of expressions included in the negotiation scenarios as the predictor variables, we conducted mathematical quantification theory class I, which is a type of multiple regression analysis. The following Eq (1) is the regression model for predicting each rating value of seven impression scales. *Y* represents the rating values of the respective 7 impression scales, and *X1*–*X3* are euphemistic expressions, honorific expressions, and sympathy expressions, respectively:

$$Y=\sum_{i=1}^{3}X_{i}+Const.$$ (1)

Table 2 summarizes the regression equation. The results show that: honorific expression is the most important factor contributing to conscientiousness; sympathy expression is the most important factor contributing to extraversion, intelligence, and emotional stability; and euphemistic expression is the most important factor contributing to positive impression, agreeableness, and degree of persuasion.

**Table 2:** Regression model predicting impression values for euphemistic, honorific, and sympathy expressions.

*Y*	Honorific expression *X1*	Euphemistic expression *X2*	Sympathy expression *X3*	Const	*R*^*2*^
Positive impression	0.506*	0.592*	0.575*	-0.692	0.993
Extraversion	0.405*	0.507*	0.536*	-0.316	0.976
Intelligence	0.499*	0.515*	0.533*	-0.523	0.990
Conscientiousness	0.607*	0.513*	0.404*	-0.468	0.993
Emotional stability	0.434*	0.435*	0.441*	-0.349	0.988
Agreeableness	0.496*	0.603*	0.590*	-0.609	0.988
Degree of persuasion	0.507*	0.593*	0.554*	-0.559	0.991

*; *p* < .01

The most contributing expression for each impression scale is highlighted in gray.

We also analyzed whether optimal linguistic expressions might be different depending on the negotiator’s appearance. First, participants evaluated their impressions obtained from the physical appearance of the people in the photographs. As shown in Tables 3–5, participants tended to give photographs of persons with good appearance the highest score for positive, extraversion, intelligence, conscientiousness, emotional stability, and agreeableness. They tended to give photographs of persons with bad appearance the lowest score for positive, extraversion, intelligence, conscientiousness, emotional stability, and agreeableness. This result is consistent with a previous study [12], which found that strangers rated attractive people as possessing socially desirable traits to a greater extent than unattractive people.

**Table 3:** Average values of impressions of each photograph.

Male photographs	Positive	Extraversion	Intelligence	Conscientiousness	Emotional stability	Agreeableness
Appearance A	1.67	2.06	0.98	0.76	0.83	1.63
Appearance B	0.25	0.53	0.6	0.69	0.41	0.32
Appearance C	-2.19	-1.61	-1.1	-1.08	-1.4	-1.84

**Table 4:** Average values of impressions of female photographs.

Female photographs	Positive	Extraversion	Intelligence	Conscientiousness	Emotional stability	Agreeableness
Appearance A	1.67	2.06	0.98	0.76	0.83	1.63
Appearance B	0.25	0.53	0.6	0.69	0.41	0.32
Appearance C	-2.19	-1.61	-1.1	-1.08	-1.4	-1.84

**Table 5:** Average values of impressions of male photographs.

Male photographs	Positive	Extraversion	Intelligence	Conscientiousness	Emotional stability	Agreeableness
Appearance A	1.56	1.98	0.9	0.92	1.11	1.81
Appearance B	0.4	0.37	0.98	0.87	0.66	0.42
Appearance C	-2.11	-1.73	-0.31	-0.4	-1.15	-1.85

By using the average rating values for the seven affective factors as the objective variables, and three language factors composing negotiation sentences (euphemistic, honorific, and sympathy expressions) as the predictor variables, we conducted mathematical quantification theory class I. We obtained the multiple regression equation indicating the importance of the three language factors (euphemistic, honorific, and sympathy expressions) for impressions made during negotiation. Tables 6–8 show the results of the analysis. In the tables, we highlighted the most important language factors for each affective scale. The results suggest that euphemistic expressions are effective for persons with relatively good appearance, while persons with bad appearance should use honorific expressions to obtain the evaluation of affective scales.

**Table 6:** Regression model predicting impression values for euphemistic, honorific, and sympathy expressions in the case of negotiators with good appearance. The most contributing expression for each impression scale is highlighted in gray.

*Y*	Honorific expression *X1*	Euphemistic expression *X2*	Sympathy expression *X3*	Const	*R*^*2*^
Positive impression	0.457*	0.680*	0.627*	-0.012	0.985
Extraversion	0.393	0.588*	0.548*	0.377	0.977
Intelligence	0.554*	0.614*	0.527*	-0.116	0.984
Conscientiousness	0.587*	0.603*	0.445*	-0.082	0.989
Emotional stability	0.503*	0.443*	0.440*	0.152	0.973
Agreeableness	0.505	0.660*	0.614*	-0.028	0.967
Degree of persuasion	0.520*	0.636*	0.601*	0.009*	0.976

*; *p* < .01

**; *p* < .001

**Table 7:** Regression model predicting impression values for euphemistic, honorific, and sympathy expressions in the case of negotiators with normal appearance. The most contributing expression for each impression scale is highlighted in gray.

*Y*	Honorific expression *X1*	Euphemistic expression *X2*	Sympathy expression *X3*	Const	*R*^*2*^
Positive impression	0.461**	0.574**	0.587**	-0.428	0.997
Extraversion	0.350*	0.486*	0.541*	-0.082	0.986
Intelligence	0.395	0.471*	0.554*	-0.256	0.969
Conscientiousness	0.548*	0.522*	0.404*	-0.281	0.992
Emotional stability	0.387**	0.450**	0.459**	-0.130	0.997
Agreeableness	0.459*	0.634*	0.627*	-0.423	0 .999
Degree of persuasion	0.439*	0.618*	0.595*	-0.341	0.987

*; *p* < .01

**; *p* < .001

**Table 8:** Regression model predicting impression values for euphemistic, honorific, and sympathy expressions in the case of negotiators with bad appearance. The most contributing expression for each impression scale is highlighted in gray.

*Y*	Honorific expression *X1*	Euphemistic expression *X2*	Sympathy expression *X3*	Const	*R*^*2*^
Positive impression	0.605*	0.492*	0.480*	-1.616	0.971
Extraversion	0.476	0.420	0.489	-1.224	0.934
Intelligence	0.550*	0.441*	0.496*	-1.181	0.993
Conscientiousness	0.688*	0.396	0.343	-1.029	0.958
Emotional stability	0.416*	0.390*	0.400*	-1.051	0.981
Agreeableness	0.527*	0.490*	0.502*	-1.358	0.975
Degree of persuasion	0.565*	0.502*	0.440*	-1.328	0.978

*; *p* < .01

**; *p* < .001

Tables 9–14 show impression differences between evaluations of photographs only and photographs with negotiation sentences in various cases. They indicate that impressions of a negotiator with a relatively good appearance might be worsened using negotiation sentences, while those of a negotiator with a bad appearance can be improved using negotiation sentences. We conducted one-way factorial ANOVA for the affective data of female and male photographs respectively (factor: photograph only or photograph with negotiation sentence). The one-way factorial ANOVA showed a significant main effect for negotiation sentences (female: F(5,19184) = 40.911, *p* < .001; male: F(5,18684) = 50.568, *p* < .001). As for appearance A of female and male photographs, multiple comparisons with Bonferroni correction showed significant differences between photograph only and photograph with negotiation (female: *p* < .05; male: *p* < .001). As for appearance B of female and male photographs, multiple comparisons with Bonferroni correction confirmed that there were no significant differences between photograph only and photograph with negotiation (female: *p* = .056; male: *p* < 1.000). In contrast, as for appearance C of female and male photographs, that is, bad appearance, multiple comparisons with Bonferroni correction showed significant differences between photograph only and photograph with negotiation (female: *p* < .001; male: *p* < .001). These results indicate that the impressions of female and male negotiators with a relatively normal appearance are not easily affected by the negotiators’ use of language, whereas the impressions of female and male negotiators with good and bad appearances can be degraded or improved by their use of language.

**Table 9:** Impression differences from photograph only and photograph with negotiation sentence in the case of female photographs with good appearance.

Good appearance Female	Positive	Extraversion	Intelligence	Conscientiousness	Emotional stability	Agreeableness
Only photograph	0.56	2.14	1.06	0.61	1.45	1.63
With negotiation	0.86	1.31	0.93	0.91	0.96	0.99

**Table 10:** Impression differences from photograph only and photograph with negotiation sentence in the case of male photographs with good appearance.

Good appearance Male	Positive	Extraversion	Intelligence	Conscientiousness	Emotional stability	Agreeableness
Only photograph	1.56	1.98	0.9	0.92	1.11	1.81
With negotiation	0.84	1.19	0.77	0.79	1.03	1.02

**Table 11:** Impression differences from photograph only and photograph with negotiation sentence in the case of female photographs with normal appearance.

Normal appearance Female	Positive	Extraversion	Intelligence	Conscientiousness	Emotional stability	Agreeableness
Only photograph	0.17	0.69	0.22	0.52	0.22	0.32
With negotiation	0.48	0.73	0.49	0.51	0.5	0.56

**Table 12:** Impression differences from photograph only and photograph with negotiation sentence in the case of male photographs with normal appearance.

Normal appearance Male	Positive	Extraversion	Intelligence	Conscientiousness	Emotional stability	Agreeableness
Only photograph	0.4	0.37	0.98	0.87	0.66	0.42
With negotiation	0.47	0.69	0.63	0.62	0.75	0.63

**Table 13:** Impression differences from photograph only and photograph with negotiation sentence in the case of female photographs with bad appearance.

Bad appearance female	Positive	Extraversion	Intelligence	Conscientiousness	Emotional stability	Agreeableness
Only photograph	-1.64	-1.5	-1.86	-1.73	-1.83	-1.84
With negotiation	-0.47	-0.39	-0.45	-0.35	-0.5	-0.49

**Table 14:** Impression differences from photograph only and photograph with negotiation sentence in the case of male photographs with bad appearance.

Bad appearance male	Positive	Extraversion	Intelligence	Conscientiousness	Emotional stability	Agreeableness
Only photograph	-2.11	-1.73	-0.31	-0.4	-1.15	-1.85
With negotiation	-0.75	-0.48	-0.22	-0.07	-0.26	-0.49

With respect to gender characteristics, the one-way factorial ANOVA of the degrees of persuasion showed no significant main effect for gender (F(3, 4115) = 1.681, *p* = .169). However, male and female gender showed the significant characteristic of optimal linguistic expressions. Tables 15 and 16 show the results of regression analysis using female participants’ responses when they evaluated expressions associated with female and male photographs. For both female and male participants, the sympathy expression is the most important factor contributing to positive impressions, while extraversion, intelligence, agreeableness, and honorific expressions contribute to conscientiousness. For female photographs, the euphemistic expression is the most important factor contributing to emotional stability. In contrast, for male participants, the euphemistic expression is the most important factor contributing to degree of persuasion. This result suggests that effective expressions might be different for women and men when making an impression of emotional stability, agreeableness, and persuasiveness.

**Table 15:** Regression model predicting impression values for euphemistic, honorific, and sympathy expressions based on the responses to female photographs. The most contributing expression for each impression scale is highlighted in gray.

*Y*	Honorific expression *X1*	Euphemistic expression *X2*	Sympathy expression *X3*	Const	*R*^*2*^
Positive impression	0.429*	0.568*	0.597*	-0.516	0.981
Extraversion	0.334	0.450*	0.543*	-0.151	0.979
Intelligence	0.526*	0.493*	0.533*	-0.547	0.990
Conscientiousness	0.596*	0.503*	0.374*	-0.453	0.984
Emotional stability	0.433*	0.444*	0.440*	-0.429	0.994
Agreeableness	0.452*	0.591*	0.604*	-0.555	0.983
Degree of persuasion	0.365	0.511*	0.587*	-0.293	0.977

*; *p* < .001

**Table 16:** Regression model predicting impression values for euphemistic, honorific, and sympathy expressions based on the responses to male photographs. The most contributing expression for each impression scale is highlighted in gray.

*Y*	Honorific expression *X1*	Euphemistic expression *X2*	Sympathy expression *X3*	Const	*R*^*2*^
Positive impression	0.531*	0.530*	0.584*	-0.760	0.990
Extraversion	0.419	0.500	0.561	-0.382	0.927
Intelligence	0.486*	0.487*	0.556*	-0.481	0.983
Conscientiousness	0.586*	0.449*	0.448*	-0.402	0.980
Emotional stability	0.405*	0.371*	0.456*	-0.198	0.977
Agreeableness	0.467	0.520	0.598*	-0.521	0.953
Degree of persuasion	0.571*	0.586*	0.565*	-0.690	0.969

*; *p* < .001

## Discussion

In this study, we investigated how visual appearance affects the persuasiveness of the negotiation process. Furthermore, we examined how the use of language according to visual appearance affects affective factors during the negotiation process and the influence of gender. As a result, photographs with good appearance resulted in a high degree of persuasion and photographs with bad appearance resulted in a low degree of persuasion. In addition, we were able to obtain a multiple regression equation indicating the importance of the three language factors (euphemistic, honorific, and sympathy expressions) to impressions made during negotiations. Considering gender characteristics, there was no significant difference in the effect of gender. However, male and female gender showed a significant characteristic of optimal linguistic expressions. In this section, we discuss the effect of visual appearance, use of language, and gender.

Considering visual appearance, [12] claims that “what is beautiful is good,” which shows that beauty is a stereotype in which physically attractive individuals are believed to possess various positive personal qualities. Empirical studies have demonstrated the effect of visual appearance; for example, several studies have shown that attractive people are more likely to be employed [41–44] and promoted [45–47]. In particular, visual appearance is reported to be a significant factor when recruiters assess candidates [48]. In this study, we demonstrated that photographs with good appearance resulted in a high degree of persuasion and photographs with bad appearance resulted in a low degree of persuasion. These findings are consistent with the notions of “beauty premium” [49] and “what is beautiful is good” [12]. Furthermore, the present study showed for the first time that first impressions due to facial attractiveness could change during the negotiation process. Specifically, our study showed that persons with good appearance could worsen their first impressions during negotiations by poor use of language, while persons with bad appearance could effectively improve their first impressions during negotiations through their good use of language, even though the final impressions of their negotiation counterpart might still be more negative than those for persons with good appearance. For example, the results of this study suggest that euphemistic expressions were effective for persons with relatively good appearance, while persons with bad appearance should use honorific expressions to improve their evaluation by affective scales. However, the impressions made by persons of normal appearance were not easily affected by their use of language.

Furthermore, the present study reconsidered gender stereotypes. Gender effects in negotiations have been reported with different results. For example, there are conflicting results that the degree of negotiation does not differ between men and women, and that men are more effective in negotiations than women [50–52]. In this study, there was no significant gender difference in negotiations. The reason why there was no gender influence in this study is thought to be the content of the negotiations. According to previous studies, men are reported to be more effective in negotiations than women, such as in salary negotiations or car purchases [52, 53]. The content of these negotiations is related to the pursuit of certain benefits. However, the contents of negotiations in this study were not for profit. Therefore, the content of the negotiations is an important factor in gender differences.

## Conclusions

In this study, we investigated the influence of visual appearance of negotiators on their personal or social impressions within the context of negotiations. We conducted psychological experiments to quantitatively analyze the relationship between visual appearance and the use of language. As a result, photographs with good appearance resulted in a high degree of persuasion and photographs with bad appearance resulted in a low degree of persuasion. In addition, we were able to obtain a multiple regression equation indicating the importance of the three language factors (euphemistic, honorific, and sympathy expressions) for impressions made during negotiations. The results show that there are optimal negotiation sentences based on various negotiation factors (such as visual appearance, use of language, and relationship to the negotiation counterpart). For example, the result suggests that persons of good appearance might worsen their impressions in negotiations using language, although their impressions were originally good, and persons of bad appearance could effectively improve their impressions in negotiations through language use even though their final impressions might be still lower than persons of good appearance. The results of our study also have implications for future studies of effective negotiation strategies.
